# Structural Transition in Few-Layer Group-IV Monochalcogenides Induced by Mechanical Forces

**DOI:** 10.1021/acsomega.6c03304

**Published:** 2026-05-19

**Authors:** Redhwan Moqbel, Krishna Ranganayakulu Vankayala, Rajesh Kumar Ulaganathan, Raman Sankar, Min-Nan Ou, Chi-Cheng Lee, Kung-Hsuan Lin

**Affiliations:** † Institute of Physics, 38017 Academia Sinica, Taipei 115201, Taiwan; ‡ Centre for Nanotechnology, 33561 Indian Institute of Technology, Roorkee 247667, India; § Department of Physics, 34886 Tamkang University, Tamsui Dist., New Taipei 251301, Taiwan; ∥ Taiwan Consortium of Emergent Crystalline Materials, Ministry of Science and Technology, Taipei 106214, Taiwan

## Abstract

Monolayers of group IV monochalcogenides are predicted to exhibit multiferroic behavior, combining both ferroelectric and ferroelastic properties at room temperature. However, the ferroelasticity of these materials has not yet been investigated experimentally. We theoretically report ferroelastic properties in few-layer SnSe, which demonstrate multiferroic behavior. Experimentally, polarization-resolved second-harmonic generation (SHG) measurements were conducted on few-layer SnSe flakes to reveal their in-plane polarization along the armchair direction. A force-induced structural transition was observed in several flakes of SnSe, as confirmed by SHG analysis. First-principles calculations elucidate the strain-dependent SHG response and suggest that the structural transition is associated with the process of ferroelastic or partial ferroelastic switching.

## Graphical abstract

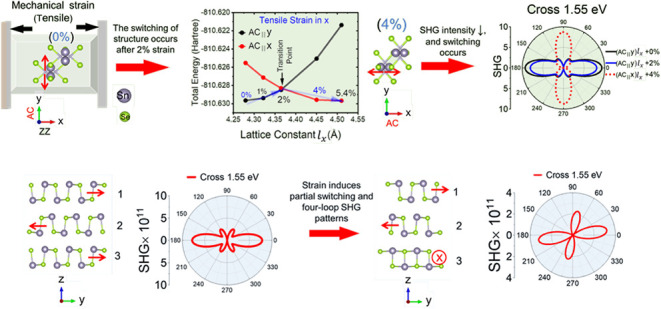

## 1 Introduction

Monolayers of group-IV monochalcogenides MX (M = Sn, Ge; X = S, Se, Te) exhibit a diverse range of characteristics, such as in-plane ferroelectricity,^1–3^ second harmonic generation (SHG),^4–6^ photostriction,^7^ large exciton binding energy,^8,9^ photovoltaics,^10,11^ electronic valley polarization, valley Hall effect,^12–14^ spin splitting of electronic bands,^15–17^ and giant spin Hall effect.^18^ MX also demonstrates outstanding physical properties, including chemical stability,^19^ high mobility,^20,21^ significant absorption coefficients,^16,22^ abundance in the Earth’s crust,^23^ nontoxicity, and environmental friendliness.^23^ The most captivating aspect lies in the potential of their monolayers to serve as 2D multiferroic substances that are theoretically predicted to exhibit both in-plane ferroelectricity and ferroelasticity.^3,24^ In-plane ferroelectricity was first confirmed in a SnTe monolayer flake using a scanning tunneling microscope (STM).^25^ Subsequently, control of ferroelectricity was demonstrated in a SnSe monolayer flake.^26^

For the bulk MX family, they typically exhibit the α-phase where the layers stack in an antiferroelectric manner (AB-stacking), leading to the disappearance of ferroelectricity. Recently, a few-layer MX with ferroelectric stacking (AC-stacking) has been discovered.^27–32^ Because the ferroelectricity is preserved across multiple layers, efficient SHG has been demonstrated.^32–34^ In contrast to ferroelectricity, there is no experimental report yet for ferroelasticity in the MX family. This scarcity may arise from the difficulty of synthesizing monolayers with large lateral sizes due to robust interlayer connections in the MX family.^35–37^ Recently, a few materials have been discovered to exhibit ferroelasticity, such as BiFeO_3_,^38^ Bi_2_WO_6_,^39^ PbTiO_3_,^40^ 1T′ MoTe_2_,^41^ ReS_2_, ReSe_2_,^42^ β’-In_2_Se_3_,^43^ and organic–inorganic perovskite.^44^ The uniqueness of the MX family is that theory predicted the existence of both ferroelectricity and ferroelasticity, leading to four ferroic states with in-plane polarizations pointing in the x, -x, y, and -y directions.^3,24^ Ferroelectric switching, where the polarization changes 180°, was experimentally demonstrated in a SnSe monolayer flake with a lateral size of 100 nm by applying voltage to an STM tip.^26^ However, strain-induced structural transitions and their possible connection to ferroelastic switching (i.e., 90° polarization rotation) have not yet been experimentally demonstrated in the MX family.

For α-phase in MX few layers, the in-plane polarization in each layer is opposite to that of the adjacent layer, leading to centrosymmetry for an even number of layers. However, for an odd number of layers, the centrosymmetry is broken, and in-plane ferroelectricity is preserved. To further induce a ferroelastic transition, it may require a strain of several percent. This necessitates a substrate capable of withstanding high strain levels. Polydimethylsiloxane (PDMS) substrates, known for their ability to sustain significant strain, have been used to apply strains of 4.6% to PbTiO_3_ thin films.^45^ In this study, we observed strain-induced structural transition in α-SnSe flakes on PDMS substrates by using polarization-resolved SHG anisotropy measurements and Raman spectroscopy. First-principles calculations elucidate the strain-dependent SHG response and suggest the structural transition results from the process of ferroelastic or partial ferroelastic switching.

## 2 Results and Discussions

### 2.1 Experimental Characterization

SnSe single crystals were synthesized via chemical vapor transport (CVT), with detailed preparation procedures provided in the experimental section. The α-phase SnSe single crystals with AB stacking were confirmed using X-ray diffraction (XRD) and pole figure XRD, as shown in Supplementary Figure S1 and Figure S2. As shown in Figure 1a, the Raman spectrum of bulk α-SnSe exhibits four distinct vibrational modes at 69.89 $\left(A_{g}^{1}\right)$ , 105.28 (*B*_3*g*_), 123.57 $\left(A_{g}^{2}\right)$ , and 148.3 $\left(A_{g}^{3}\right)$ cm^–1^. Polydimethylsiloxane (PDMS) substrates were employed for the mechanical exfoliation of the α-SnSe crystals, yielding multiple SnSe flakes with nanometer-scale thicknesses on the PDMS surfaces. Figure 1b presents the AFM image of several exfoliated SnSe flakes, showing that the lateral size generally decreases with reduced thickness. The thicknesses of three representative flakes, highlighted by white circles and labeled as the first, second, and third samples, are approximately 740, 280, and 189 nm, respectively. Figure 1c displays the Raman spectra of these three flakes. The Raman spectrum of the 740 nm-thick flake in Figure 1c shows similar features to bulk SnSe in Figure 1a with four distinct vibrational modes. However, for the thinner flakes, such as the 189 nm sample, only the $A_{g}^{1}$ mode at ∼68 cm^–1^ can be clearly identified.

**Figure 1:**
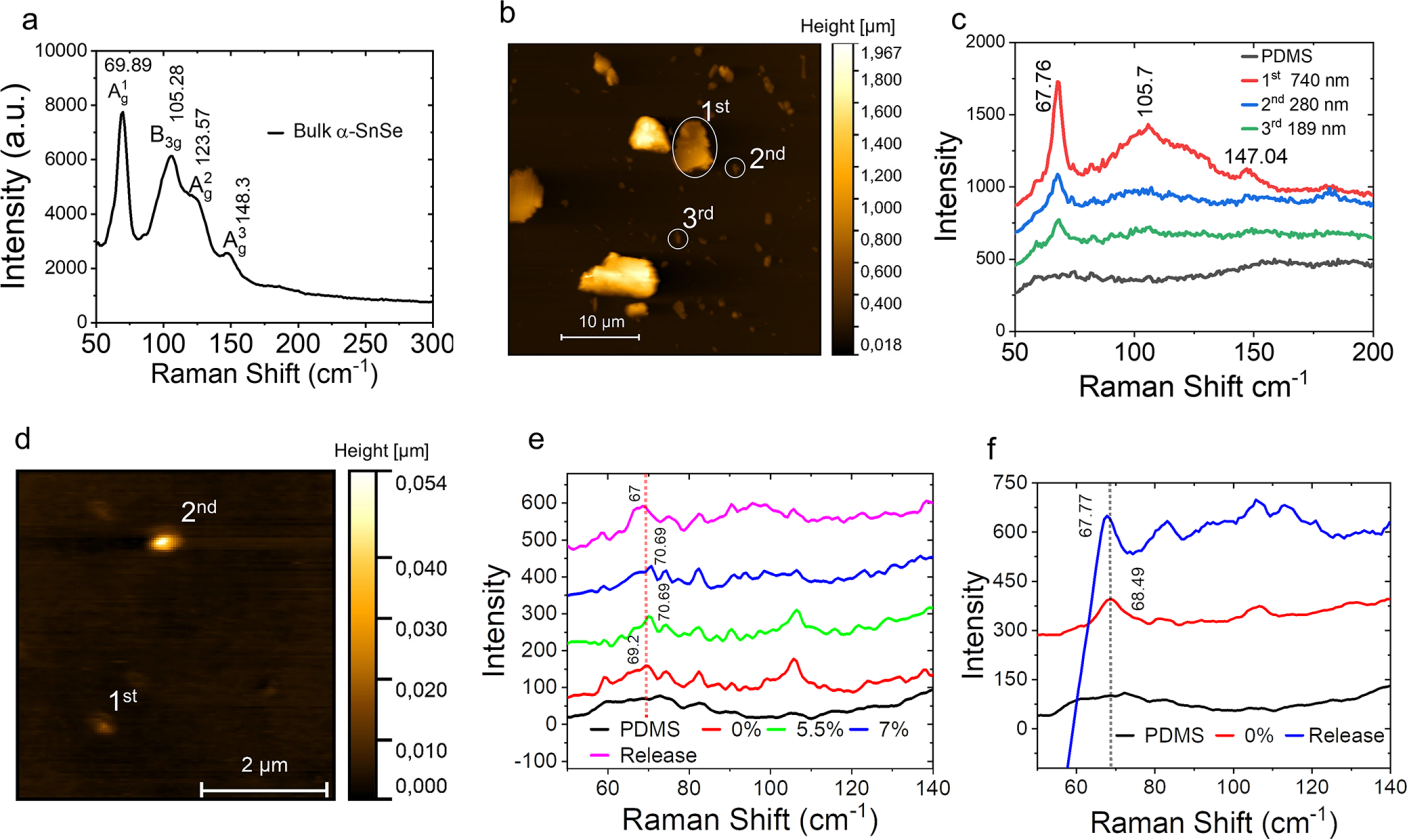
Experimental characterization of SnSe flakes. (a) Raman spectrum of a bulk α-SnSe flake. (b) AFM image of several SnSe flakes. (c) Raman spectra of the three SnSe flakes labeled in the AFM image (b). (d) AFM image of thinner SnSe flakes. Strain-dependent Raman spectra of the (e) 1^st^ and (f) 2^nd^ flakes labeled in (d).

Figure 1d shows the AFM image of two flakes with thicknesses of 11 and 46 nm, whose Raman spectra are given in Figure 1e and f, respectively. For the 11 nm-thick flake [Figure 1e], tensile strain induced by the PDMS substrate shifts the $A_{g}^{1}$ mode from 69.2 cm^‑1^ (0%) to 70.69 cm^–1^ (7%). Details of evaluating the strain exerted on SnSe by the PDMS substrate are provided in the Supporting Information. The estimated strain may be slightly overestimated. After releasing the substrate strain, the peak shifts to 67 cm^–1^, indicating that the flake does not fully recover its original state. This effect is even cleaer for the thicker flake [Figure 1f]: its unstrained $A_{g}^{1}$ mode appears at 68.49 cm^–1^, but after applying and releasing 7% strain, it shifts to 67.77 cm^–1^.

Exfoliated flakes with micron-scale lateral sizes are generally thick due to the strong van der Waals interactions between adjacent SnSe layers.^46^ When the thickness decreases below 100 nm, the lateral size typically reduces to the submicron scale. However, detecting Raman signals becomes challenging for flakes only a few hundred nanometers in lateral size and thinner than 10 nm. To overcome this limitation, we employed SHG microscopy to probe few-layer SnSe flakes. SHG is theoretically forbidden in bulk α-SnSe crystals because of their centrosymmetric structure, but thin flakes with an odd number of layers (e.g., one, three, or five layers) or thick flakes with wedding cake structures^47^ break this symmetry, thereby allowing SHG. Leveraging this property, SHG microscopy was used to raster scan the PDMS substrate and rapidly identify SnSe flakes capable of efficiently emitting SHG photons.

Figure 2a presents an atomic force microscopy (AFM) image of an exfoliated SnSe flake on a PDMS substrate, with a measured thickness of approximately 3 nm. Given that each monolayer is ∼0.6 nm thick,^48,49^ this corresponds to a five-layer SnSe flake. The same flake was examined using polarization-resolved SHG microscopy at a wavelength of 800 nm (1.55 eV). SHG images with polarization parallel and cross to the incident light polarization were recorded as functions of the sample‘s azimuthal angle. For example, Figure 2b shows the cross-polarized SHG image of the five-layer SnSe flake at a rotational angle of 180°. The angle-resolved cross-polarized and parallel-polarized SHG intensity polar patterns are shown by black lines in Figure 2c and d, respectively. The SHG pattern reveals that the flake remains AB-stacking after exfoliation, according to our theoretical calculation presented later. The crystal orientations, such as zigzag (ZZ) and armchair (AC) directions, were identified based on the SHG polar patterns. The crystal orientation is labeled in Figure 2b, where the azimuthal angle 0°/180° is defined when the ZZ direction is parallel to the laser polarization (indicated by the red double arrow).

**Figure 2:**
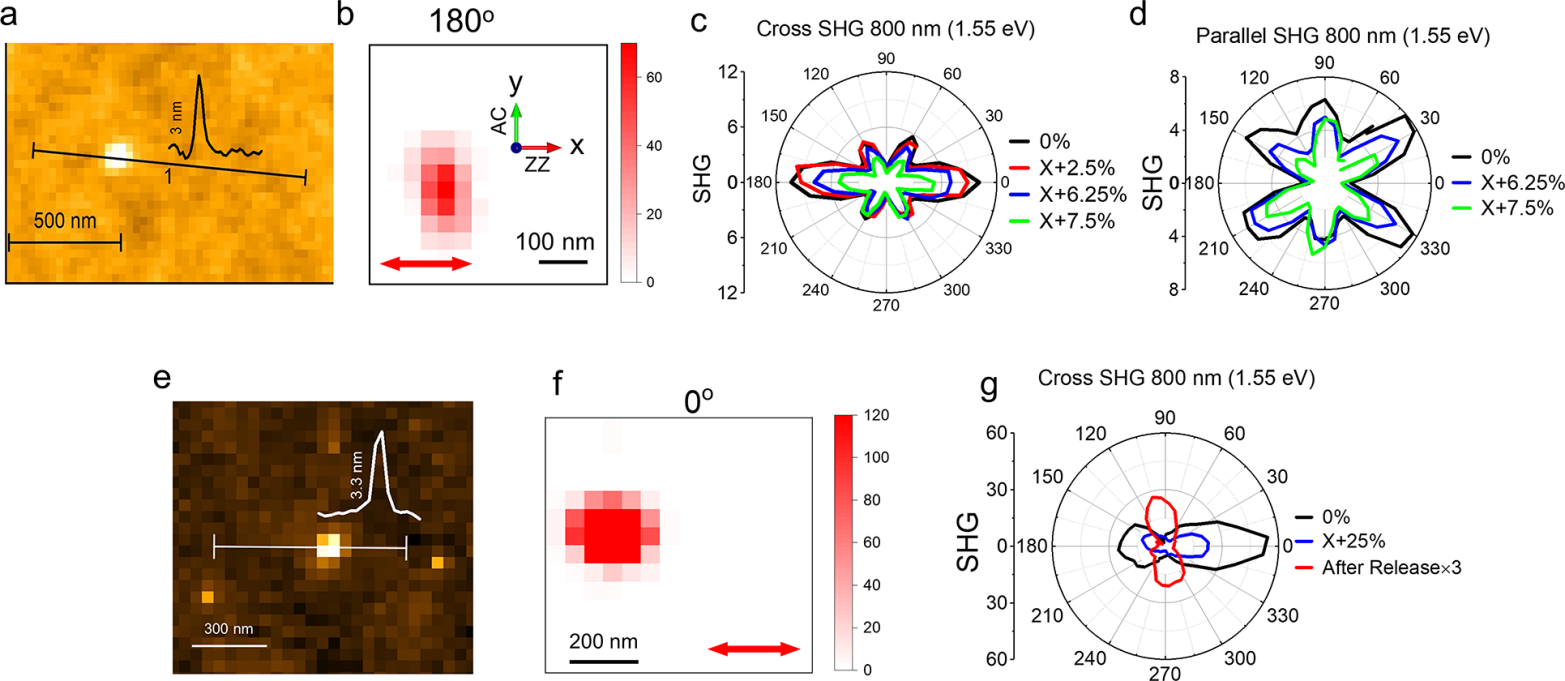
Experimental characterization of few-layer SnSe flakes. (a) AFM image of a SnSe flake on a PDMS substrate. (b) SHG image of the same SnSe flake in (a), recorded at a sample rotational angle of 180°. The red arrow indicates the polarization direction of the incident laser. (c) Cross-polarized and (d) parallel-polarized SHG intensity of the flake in (b) as a function of rotational angle. The different colors correspond to SHG polar patterns under various tensile strains applied along the *x*-axis of the SnSe. (e) AFM image of another SnSe flake on a PDMS substrate. (f) SHG image of the SnSe flake in (e), acquired at a sample rotational angle of 0°. (g) Cross-polarized SHG intensity of the flake in (f) as a function of rotational angle. The red line indicates the SHG intensity (scaled by a factor of 3) after the strain is released.

A tensile strain along the ZZ direction of the SnSe flake was applied using the PDMS substrate. The red line in Figure 2c illustrates the cross-polarized SHG polar pattern under a 2.5% strain applied along the ZZ direction of the SnSe flake. This pattern shows minimal change compared to the black line representing the unstrained state. However, when tensile strains of 6.25% and 7.5% are applied, the SHG intensity at 0° decreases by 24.4% and 50.5%, respectively. A similar trend is observed in the parallel-polarized SHG polar patterns shown in Figure 2d. At tensile strains of 6.25% and 7.5%, the SHG intensity of the lobe near 30° decreases by 22.4% and 49.4%, respectively. Note that the refractive index of PDMS is strain-dependent; tensile strain increases the refractive index of PDMS,^50,51^ which would theoretically enhance the reflectivity and SHG intensity. Consequently, the observed reduction in SHG intensity is confirmed to originate from the SnSe under tensile strain rather than from strain-dependent optical properties of the PDMS substrate.

We conducted another set of experiments to further investigate if the SHG response recovers after the release of strain. The AFM image in Figure 2e indicates a thickness of 3.3 nm. Considering that SHG is only efficient for odd-layer SnSe, the layer number should be 5 (∼3 nm) rather than 3 (∼1.8 nm) or 7 (∼4.2 nm). The thickness deviation of 0.3 nm from the 5 layers thickness of ∼3 nm might arise from several experimental factors, including tip–sample interaction effects, surface adsorbates, substrate roughness, and possible thin surface oxide layers. Figure 2f presents its cross-polarized SHG image at a rotation angle of 0°, while the angle-resolved SHG polar pattern before strain is shown by the black curve in Figure 2g. The SHG polar pattern, dominated by the two-lobe patterns along 0°/180°, is similar to the SnSe flake in Figure 2c. However, the SHG polar patterns are very sensitive to structural changes. The slight differences observed in the SHG polar patterns (in the black lines of Figure 2c and g) may result from the introduced strain after the exfoliation process by using the PDMS. Similarly, the SHG intensity (depicted by the blue curve in Figure 2g) decreases by 54% upon a tensile strain of 6.25% along the ZZ direction of the SnSe. However, we additionally measured the SHG polar pattern after the strain in the PDMS substrate was released. Interestingly, the SHG polar pattern rotates 90°, as revealed by the red curve in Figure 2g. Such a rotation suggests a strain-induced structural transition, which is consistent with the characteristics expected for ferroelastic switching, as will be discussed in conjunction with theoretical analysis.

In addition to few-layer SnSe flakes, we observed that some thick flakes (exceeding 100 layers) efficiently emit SHG photons, while most do not. These SnSe flakes, exhibiting a characteristic wedding-cake configuration, show strong SHG signals consistent with previous observations in SnS,^47^ thereby enabling further investigation of strain-induced structural transitions using SHG microscopy. Figure 3a shows the AFM topography of a representative SnSe flake with a thickness of ∼300 nm, exhibiting a characteristic “wedding-cake” structure, enabling SHG signals. The corresponding SHG image at 0° is presented in Figure 3b. Figures 3c and d display the SHG polar patterns recorded before and under tensile strain applied along the *x*-axis (zigzag direction), respectively. Upon applying ∼8% tensile strain, the SHG polar pattern undergoes a marked transformation, with a slight reduction in overall intensity. After strain release, the polar pattern evolves from the initial two-lobed structure into a four-lobed configuration. Notably, the high-intensity lobes are rotated by ∼60° relative to their original orientation. When ∼6% tensile strain is reapplied, the four-lobed pattern becomes more pronounced, though accompanied by further intensity reduction. After several strain–release cycles, the SHG polar pattern consistently stabilizes into a four-lobed structure, with preferential enhancement of the lobes oriented at ∼45° and ∼215°, as shown in Figure 3e. The Raman $A_{g}^{1}$ mode at ∼67 cm^–1^ in Figure 3f indicates the flake is still SnSe after strain release. However, the SHG pattern evolves from a two-lobe pattern (in Figure 3c) to a four-lobe pattern (in Figure 3e) after strain-release cycles, indicating that SnSe undergoes structural changes. To understand and gain deeper physical insight into the experimental results, density functional theory (DFT) was employed to perform first-principles calculations and associate SHG polar patterns with structural changes.

**Figure 3:**
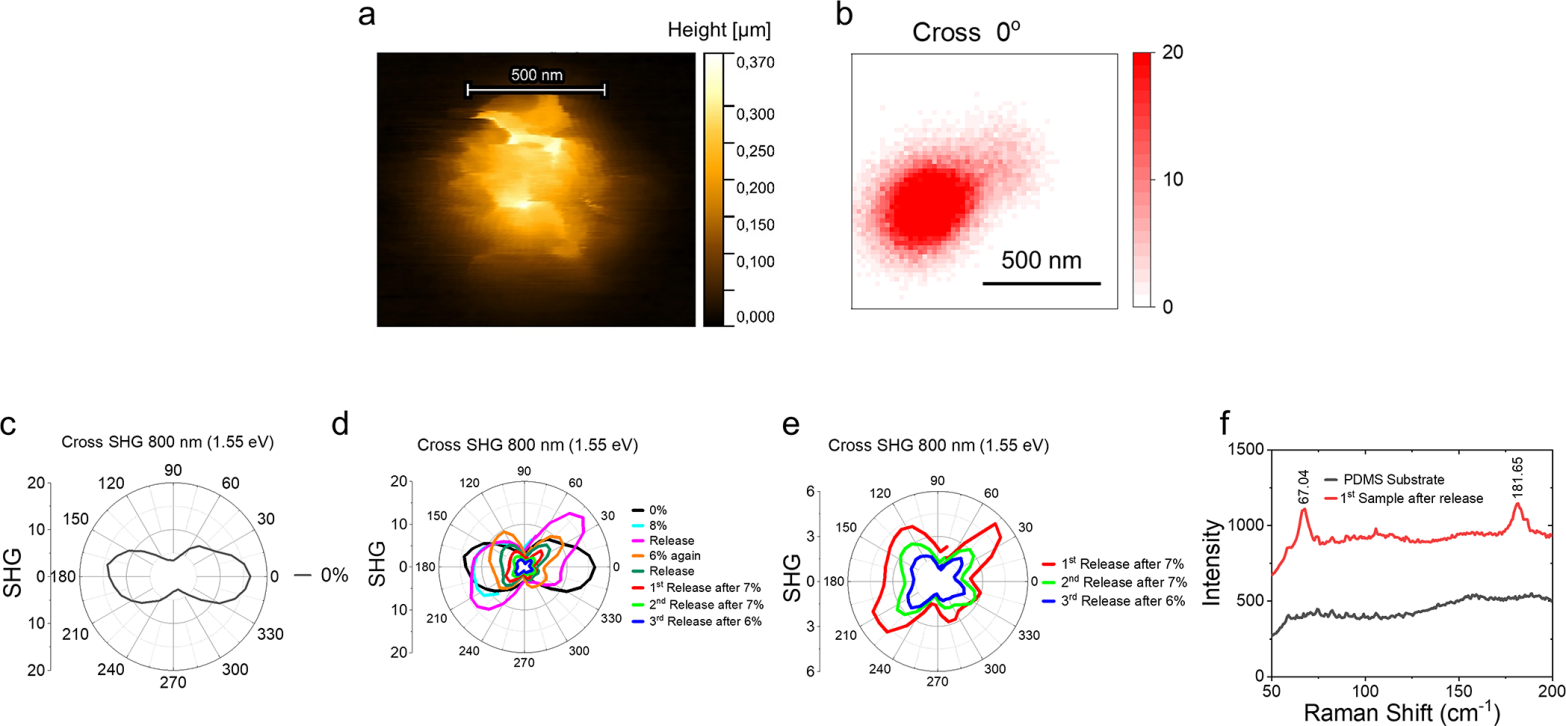
Experimental characterization of SnSe thick flakes. (a) AFM image of the SnSe flake on the PDMS substrate. (b) SHG images taken at an angle of 0°. Cross SHG polar patterns are shown (c) before strain and (d) after applying varying levels of tensile strain along the *x*-axis (zigzag) and release. (e) Cross SHG polar patterns are shown after release from several strains in the *x*-axis. (f) Raman spectrum of the SnSe flake.

### 2.2 Theoretical Analysis

We first consider a five-layer AB-stacking SnSe (which corresponds to the experimental results in Figure 2) in a relaxed form, as shown in Figure 4a. The lattice constants are *l_x_* = 4.279 Å and *l_y_* = 4.510 Å. The dipole moments are along the AC direction, as shown by the red arrows. Here, we denote this orientation as (AC_∥_y), where AC is along the *y*-axis. A reversible strain is defined as (*l_y_* – *l_x_*)/*l_x_*, which is 5.4% in this case. This means that when a uniaxial strain stretches *l_x_* to the length of *l_y_*, the relaxed structure with minimum energy should be equivalent to a 90° rotation of the original structure, as shown in Figure 4c. In this orientation, the AC is along the *x*-axis, denoted (AC_∥_x) here. The transformation between these two orientations represents the switching between two ferroelastic states.

**Figure 4:**
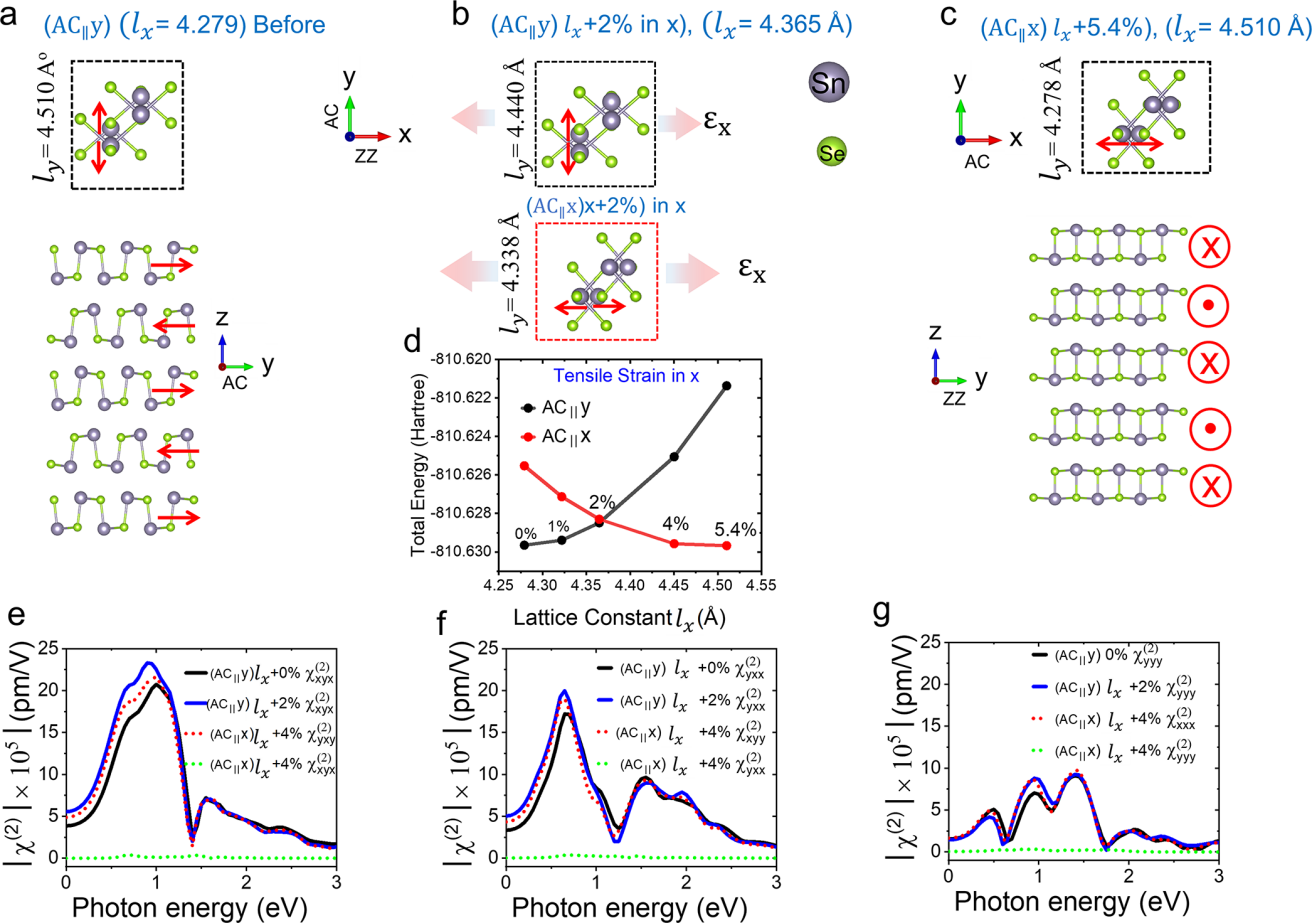
Theoretical characterization of five-layer SnSe with AB stacking. (a) Top and side views of the strain-free structure for the state (AC_∥_y). (b) Top views of the structures for the states (AC_∥_y) and (AC_∥_x), respectively under the strain condition with a fixed lattice constant along x with *l_x_* = 4.365*Å*. (c) Top and side views of the strain-free structure for the state (AC_∥_x). (d) Total energy of the structures of (AC_∥_y) and (AC_∥_x) as a function of *l_x_*. The absolute values of SHG susceptibility spectra of (e) $\chi_{xyx}^{\left(2\right)}\left(\chi_{yxy}^{\left(2\right)}\right)$ , (f) $\chi_{yxx}^{\left(2\right)}\left(\chi_{xyy}^{\left(2\right)}\right)$ , (g) $\chi_{yyy}^{\left(2\right)}\left(\chi_{xxx}^{\left(2\right)}\right)$ under different strain conditions.

The transition path from one ferroelastic state to another can be explored by calculating the total energy as a function of strain. The minimum energy barrier that must be overcome corresponds to the transformation strain, which marks the point where the two ferroelastic states become energetically degenerate. Typically, transition pathways are obtained via NEB calculations.^24^ However, we calculated the fully relaxed structure at varying lattice constants, which could be closer to experimental conditions. We relaxed the structures of the two ferroelastic states (AC_∥_y) and (AC_∥_x) by fixing the lattice constant *l_x_*, which is in accordance with the fact that strain is a controlling factor in experiments. The total energies against *l_x_* for the two states are shown in Figure 4d, respectively. The black dots reveal the state (AC_∥_y), and the minimum energy occurs at *l_x_* = 4.279*Å*. When *l_x_* increases by 1%, 2%, 4%, and 5.4%, the energy increases monotonically. On the other hand, the total energy of the (AC_∥_x) state, as depicted by red dots, is minimum at the reversible strain of 5.4% (*l_x_* = 4.510*Å*). The energy increases with decreasing *l_x_*. The crossing point of these two curves, which is called the transformation strain, is at approximately *l_x_* = 4.365 Å (corresponding to a tensile strain of 2%). Figure 4b reveals the structures of the two states (AC_∥_y) and (AC_∥_x) with degenerate total energy under the fixed *l_x_*. The transition between (AC_∥_y) and (AC_∥_x) states involves the variation of *l_y_* and corresponding atomic displacements. Ideally, when the uniaxial tensile strain on the (AC_∥_y) state (black dots in Figure 4d) exceeds the transformation strain, it prefers to transform into the (AC_∥_x) state (red dots in Figure 4d), which has lower total energy. Ferroelastic switching could theoretically occur right after the transformation strain, which is smaller than the reversible strain. The (AC_∥_y) states at 4% and 5.4% are difficult to maintain in their structures due to having higher total energies than those of the (AC_∥_x) states

In order to obtain the SHG polar patterns for comparison with the experimental results, we further calculated the SHG susceptibilities of the structures considered in Figure 4. Since the structure of α-SnSe with an odd number of layers belongs to the C_2v_ point group, it leads to seven nonzero tensor elements of SHG susceptibility: $\chi_{yxx}^{\left(2\right)},\chi_{yyy}^{\left(2\right)},\chi_{yzz}^{\left(2\right)},\chi_{xyx}^{\left(2\right)}=\chi_{xxy}^{\left(2\right)},\chi_{zzy}^{\left(2\right)}=\chi_{zyz}^{\left(2\right)}$ for the coordinate of the state (AC_∥_y). For the other state $({\mathrm{AC}}_{∥}x)$ , which is a 90° rotation of (AC_∥_y), the nonzero elements of SHG susceptibility are $\chi_{xyy}^{\left(2\right)},\chi_{xxx}^{\left(2\right)},\chi_{xzz}^{\left(2\right)},\chi_{yxy}^{\left(2\right)}=\chi_{yyx}^{\left(2\right)},\chi_{zzx}^{\left(2\right)}=\chi_{zxz}^{\left(2\right)}$ , where *x* ↔ *y*. Since the incident and collected SHG light are both linearly polarized in the xy plane in our experimental geometry, Figure 4e–g only presents the nonzero SHG susceptibilities without the z components $\left(\chi_{yxx}^{\left(2\right)},\chi_{yyy}^{\left(2\right)},\chi_{xyx}^{\left(2\right)}\right)$ or $\left(\chi_{xyy}^{\left(2\right)},\chi_{xxx}^{\left(2\right)},\chi_{yxy}^{\left(2\right)}\right)$ . The complete SHG susceptibility spectra are presented in Supplementary Figure S8. The black and blue lines in Figure 4e–g display the SHG susceptibility spectra of $\chi_{xyx}^{\left(2\right)},\chi_{yxx}^{\left(2\right)},\chi_{yyy}^{\left(2\right)}$ for strain 0% and 2%, respectively, where the (AC_∥_y) state is stable. While the strain is 4%, (AC_∥_x) becomes the preferable state instead. $\chi_{xyx}^{\left(2\right)},\chi_{yxx}^{\left(2\right)},\chi_{yyy}^{\left(2\right)}$ drop to zero, as shown by the green dotted lines. Instead, the nonzero terms are $\chi_{yxy}^{\left(2\right)},\chi_{xyy}^{\left(2\right)},\chi_{xxx}^{\left(2\right)}$ for (AC_∥_x) as shown by the red dotted lines, and their values are comparable to the nonzero terms of (AC_∥_y).

To plot the SHG polar patterns, the SHG susceptibility tensor, reduced by disregarding the z components, can be represented as follows:

1 $$\chi^{\left(2\right)}=\left(\begin{array}{lll} \chi_{xxx}^{\left(2\right)} & \chi_{xyy}^{\left(2\right)} & \chi_{xyx}^{\left(2\right)}\\ \chi_{yxx}^{\left(2\right)} & \chi_{yyy}^{\left(2\right)} & \chi_{yyx}^{\left(2\right)} \end{array}\right)$$

Suppose the linear polarization angle of the incident light is θ relative to the *x*-axis. The susceptibilities for SHG with parallel (cross) polarization to the incident polarization, denoted as $\chi_{∥}^{\left(2\right)}$ $\left(\chi_{⊥}^{\left(2\right)}\right)$ are determined using appropriate Euler angles as follows:

2 $$\left(\begin{array}{l} \chi_{∥}^{\left(2\right)}\\ \chi_{⊥}^{\left(2\right)} \end{array}\right)=\left(\begin{array}{ll} \cos\theta & \sin\theta \\ -\sin\theta & \cos\theta \end{array}\right)\chi^{\left(2\right)}\left(\begin{array}{l} \cos^{2}\theta \\ sin^{2}\theta \\ 2\cos\theta \sin\theta \end{array}\right)$$

Therefore, the angle-dependent SHG susceptibilities for (AC_∥_y) are:

3 $$\chi_{∥}^{\left(2\right),({\mathrm{AC}}_{∥}y)}\left(\theta \right)=\left(2\chi_{xyx}^{\left(2\right)}+\chi_{yxx}^{\left(2\right)}\right)\sin\;\theta \;cos^{2}\theta +\chi_{yyy}^{\left(2\right)}sin^{3}\theta$$

and

4 $$\chi_{⊥}^{\left(2\right),({\mathrm{AC}}_{∥}y)}\left(\theta \right)=\chi_{\mathrm{yxx}}^{\left(2\right)}\cos^{3}\theta +(\chi_{\mathrm{yyy}}^{\left(2\right)}-{2\chi }_{\mathrm{xyx}}^{\left(2\right)})\cos{\theta \sin}^{2}\theta$$

The angle-dependent SHG susceptibilities for (AC_∥_x) are:

5 $$\chi_{∥}^{\left(2\right),({\mathrm{AC}}_{∥}x)}\left(\theta \right)=\left(2\chi_{yxy}^{\left(2\right)}+\chi_{xyy}^{\left(2\right)}\right)\cos\theta sin^{2}\theta +\chi_{xxx}^{\left(2\right)}cos^{3}\theta$$

and

6 $$\chi_{⊥}^{\left(2\right),({\mathrm{AC}}_{∥}x)}\left(\theta \right)=-\chi_{\mathrm{xyy}}^{\left(2\right)}\sin^{3}\theta -\left(\chi_{\mathrm{xxx}}^{\left(2\right)}-2\chi_{\mathrm{yxy}}^{\left(2\right)}\right)\sin{\theta \;\cos}^{2}\theta$$

Figure 5 reveals the calculated SHG polar patterns at photon energies of 0.7, 1.4, and 1.55 eV. The patterns are energy-dependent due to the relative strength of SHG susceptibilities varying as a function of photon energy, as shown in Figure 4e–g. The strain-induced effects on the SHG intensity can also differ dramatically for different energies. For example, as shown in Figure 5a and b, the SHG intensity of 2% strain (in blue lines) is higher than that of 0% strain (in black lines) at 0.7 eV. However, at 1.55 eV for Figure 5e and 5f, the increased strain reduces the SHG intensity. Finally, the dotted lines depict the patterns of (AC_∥_x) while the solid lines display the patterns of (AC_∥_y). The patterns manifest a 90° rotation between these two ferroelastic states, which is especially clear in Figure 5d and e.

**Figure 5:**
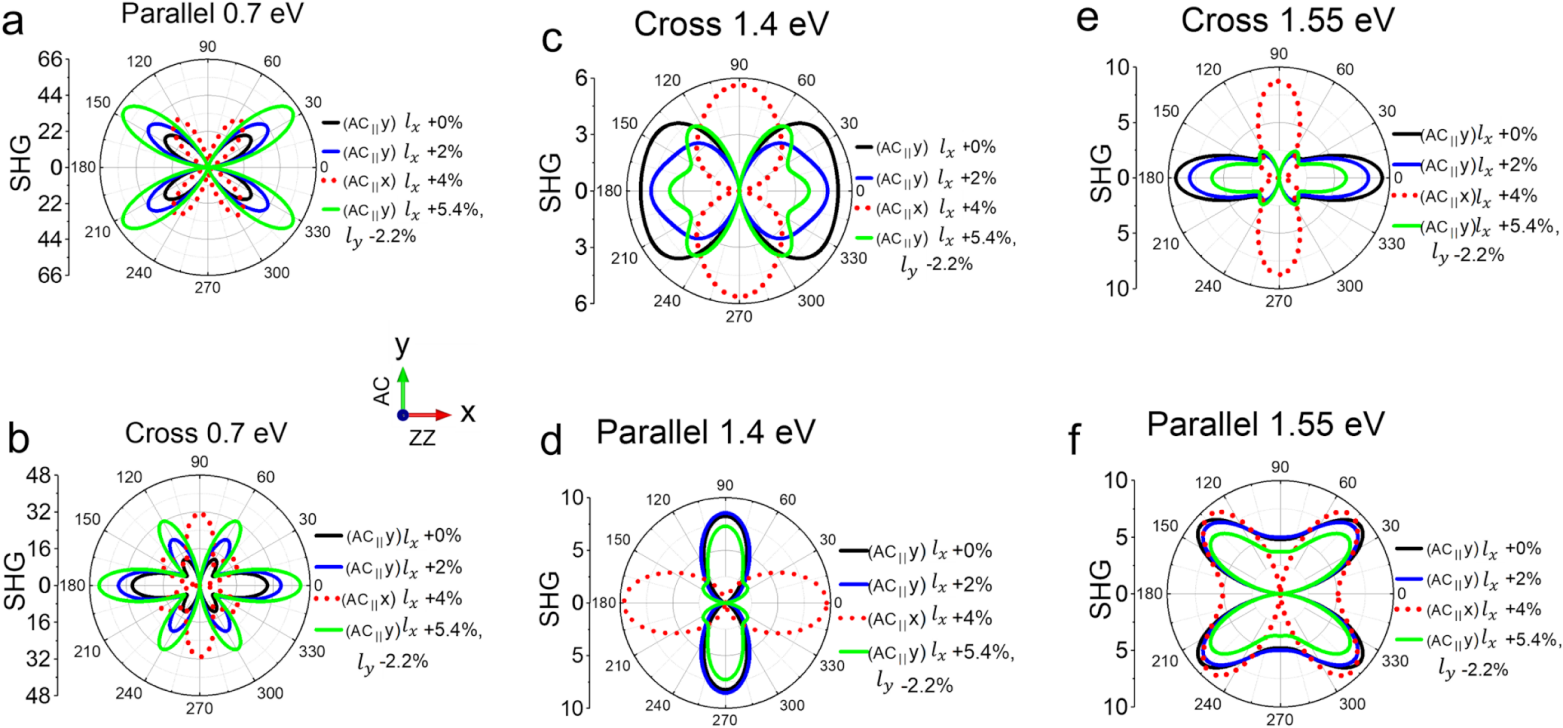
Calculated SHG polar patterns of five-layer SnSe with AB stacking. **(**a, c, e) Cross-polarized and (b, d, f) parallel-polarized SHG polar patterns at 0.7, 1.4, and 1.55 eV, respectively, under different conditions of strain. Solid lines depict the curves for the state (AC_∥_y) while dotted lines display the patterns for the state (AC_∥_x).

### 2.3 Layer-Dependent Transformation Strain

Figure 4 considers applying uniaxial tensile strain along the x of a 5-layer SnSe to transform (AC_∥_y) into the (AC_∥_x) state. It is also feasible to apply uniaxial compressive strain along y to switch from (AC_∥_y) to the (AC_∥_x). We calculated the relaxed structures of the two states as a function of a fixed *l_y_*. Figure 6a reveals the total energy of the structure, and the compressive transformation strain occurs at 3.13%. Similar calculations were conducted for 3-layer and monolayer SnSe. Details of the calculated results are provided in Figures S10–S15. Figure 6c summarizes the transformation strain as a function of the layer number. The tensile transformation strain is overall lower than the compressive one. Additionally, the transformation strain increases as the number of layers increases.

**Figure 6:**
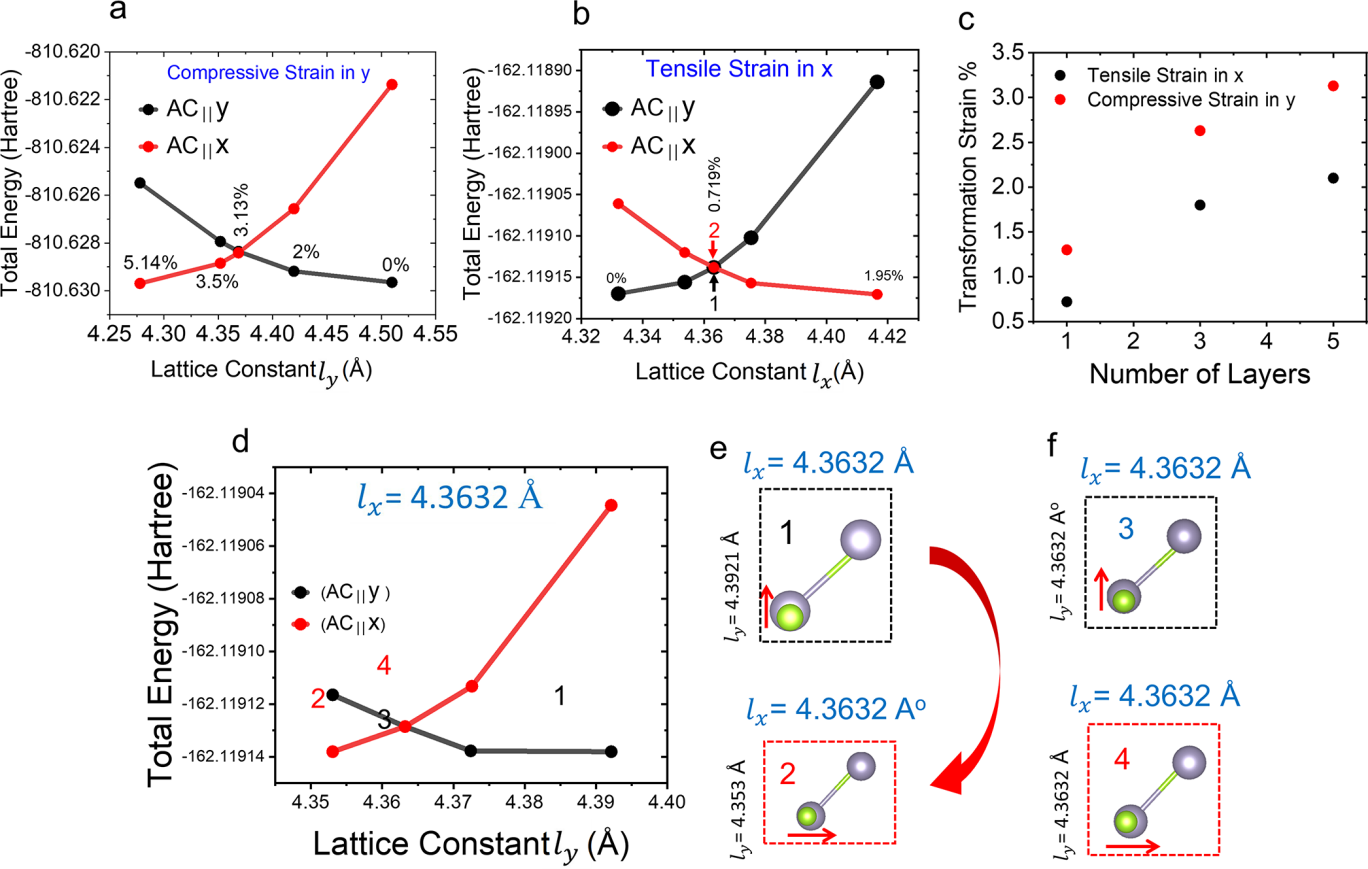
Transformation strain of ferroelastic switching. (a) Total energy of five-layer AB-SnSe in different states as a function of *l_y_*. (b) Total energy of the structures of monolayer SnSe as a function of *l_x_*. (c) Transformation strain as a function of layer number. (d) Total energy of monolayer SnSe in different states as a function of *l_y_* under a fixed *l_x_* at the transformation strain. (e) Top view of the SnSe monolayer structures corresponding to the labeled numbers in (b) and (d). The transition path from 1 to 2 goes through 3 and 4.

As shown in Figure 4b, the transition path from one state to another state at the transformation strain involves the variation of *l_y_* and the movement of the atoms. To understand the behavior of this transition, we further investigated strained monolayer SnSe since its computation is much more efficient compared with five-layer SnSe. Figure 6b shows the total energy of (AC_∥_y) and (AC_∥_x) of the SnSe monolayer as a function of *l_x_* (tensile strain in x). The tensile transformation strain, where the total energy for the two states is the same, is approximately 0.7193%, corresponding to *l_x_* = 4.3632 Å. The unit cells of (AC_∥_y) and (AC_∥_x) at the transformation strain are shown by labels 1 and 2 in Figure 6e. Under the fixed uniaxial strain in x, the transition from (AC_∥_y) to (AC_∥_x) goes through the pathway that *l_y_* decreases from 4.3921 Å to 4.3632 Å. Figure 6d shows the total energy of (AC_∥_y) and (AC_∥_x) of the SnSe monolayer for *l_y_* within this range. The crossing points labeled as numbers 3 and 4, where the two states have the same total energy, exhibit a square unit cell (*l_x_* = *l_y_*) as shown in Figure 6e. The polarization, induced by the vertically aligned nearest Sn and Se atoms (labeled as 3), can easily rotate 90 degrees, and the nearest Sn and Se atoms turn into a horizontally aligned configuration (labeled as 4). At the transformation strain, the transition pathway of ferroelastic switching 1→3→4→2 requires almost no energy and can be considered as having no barrier.

### 2.4 Comparison between Experiments and Calculations

The photon energy for the experiments is 1.55 eV. The cross-polarized SHG polar pattern in Figure 2c exhibits a feature of a horizontal two-lobe, which is similar to the calculated pattern in Figure 5e. When a tensile strain is applied along the ZZ direction, the horizontal two-lobe decreases much more significantly than the other four lobes, which also aligns well with the characteristics of the calculated pattern. Quantitatively, the experimental SHG intensity of the horizontal lobes decreases by 50% when a tensile strain of 7.5% is applied along the ZZ direction. For calculations, the maximum strain we consider is 5.4% tensile strain along the *x*-axis and 2.2% compressive strain along the *y*-axis. We consider biaxial strain because a uniaxial strain on the PDMS leads to a Poisson effect, resulting in approximately 40% compressive strain in the transverse direction. A 35% decrease in SHG intensity of the horizontal lobes (green line in Figure 5e) closely aligns with our experimental findings. The strain mainly reduces $\chi_{\mathrm{yxx}}^{\left(2\right)}$ since the term $\chi_{\mathrm{yxx}}^{\left(2\right)}\cos^{3}\theta$ in eq 4 corresponds to the horizontal two-lobe pattern in Figure 5e. A comparison of calculated SHG susceptibilities $\chi_{xyx}^{\left(2\right)},\chi_{yxx}^{\left(2\right)},\chi_{yyy}^{\left(2\right)}$ at 1.55 eV between unstrained and strained AB-SnSe five layers is referred to Figure S9.

In terms of the total energy, the transformation strain and reversible strain are ∼2% and 5.4%, respectively. Ideally, ferroelastic switching can occur when the strain is larger than the transformation strain. However, the experimental results indicate that it remains in the original ferroelastic state rather than switching into another ferroelastic state when the strain consistently increases to 7.5%. After the strain in the PDMS substrate is released, the SHG polar pattern manifests a 90° rotation in the experiments (Figure 2g), which is consistent with the calculated result of ferroelastic switching in Figure 5e. The substrate constraints quenching the ferroelastic switching were also observed in the literature,^40^ which may explain why it remains in the same ferroelastic state up to high strain. However, during the process of releasing strain, slippage may occur and minimize the constraints of the PDMS substrate on the SnSe flake, leading to a structural transition. The presence of the PDMS substrate may be needed in the theoretical simulations to better account for the experimental results. In addition, the calculations do not take into account the effect of entropy in the free energy, which contributes at room temperature. The current theoretical result considers the total energy for a fixed strain, which corresponds to the Helmholtz free energy at 0 K. Since ferroelastic switching occurs immediately after the strain is removed, the dynamics may go beyond the quasi-static regime. We leave such an analysis to future work.

For the experimental results of thicker flakes, the SHG polar patterns evolved from their initial two-lobe pattern to four-lobe (Figure 3c and e) after the PDMS substrate was strained and released. This behavior differs from that of 5-layer SnSe (Figure 2g), in which the pattern is simply rotated by 90° from the initial two-lobe pattern. We attribute the result of 5-layer SnSe to complete ferroelastic switching, where the in-plane polarization of all layers rotates by 90°. By contrast, partial ferroelastic switching occurs in thicker flakes, where only a portion of the layers (likely near the bottom) rotate by 90°. To support this argument, we performed simulations of partial layer switching in three layers of SnSe (Figure 7), as simulating thicker systems is computationally demanding and requires significantly longer runtimes. Figure 7d shows the calculated SHG polar response of AB-stacked three-layer SnSe, exhibiting two dominant lobes at 0° and 180°. When layer number three is rotated by 90°, as illustrated in Figure 7f and g, the simulated SHG polar response transforms into a four-lobed pattern, with two lobes more intense than the other two (Figure 7h). This simulated response is in excellent agreement with our experimental results in Figure 3e, providing support for the interpretation of partial ferroelastic switching in α-SnSe. Similar phenomena of partial switching were observed in another two flakes, as shown in the Supplementary Information. Additionally, the Raman peaks in Figure 1e and f show blue-shifts in the strain-released samples compared with their initial states, providing further evidence of structural changes.

**Figure 7:**
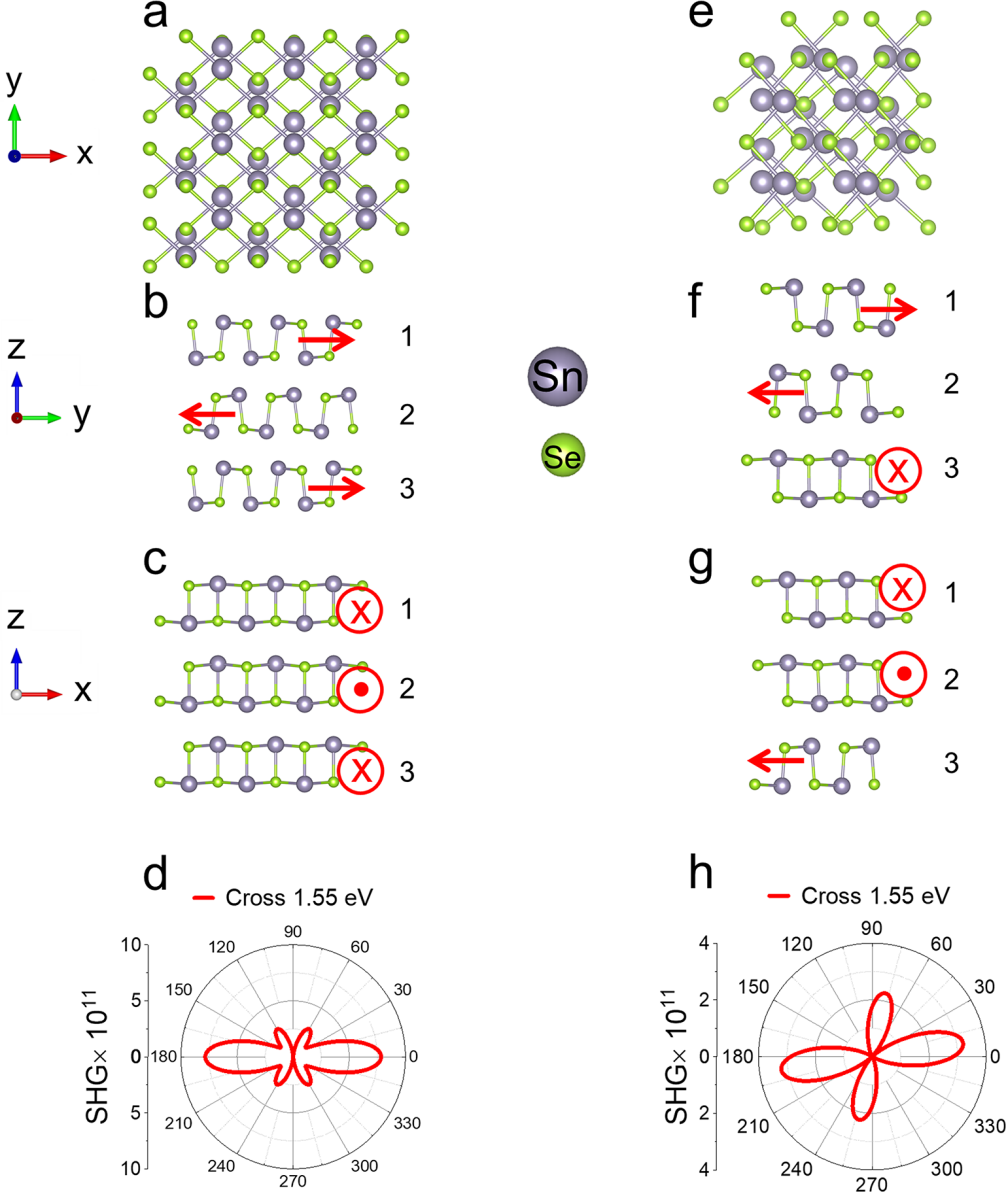
SHG polar patterns of AB-stacked SnSe and partially switched three-layer SnSe. (a–c) Top and side views of AB-stacked three-layer SnSe. (d) Cross-polarized SHG patterns at 1.55 eV for AB-stacked three layers. (e–g) Top and side views of three-layer SnSe with the third layer rotated by 90°. (h) Cross-polarized SHG patterns of three-layer SnSe with partial switching, where the third layer is rotated by 90°.

## 3 Conclusions

α-phase is the most common and stable phase for bulk group-IV monochalcogenides (MX). Among the MX family, SnSe has the minimum ferroic switching energy. We reported that α-phase SnSe few-layers with an odd-number layer exhibit multiferroic properties via a theoretical approach. The calculated total energy suggests that the transformation strain increases with increasing layer number. Experimentally, we observed a force-induced structural transition in a 5-layer SnSe flake, as well as in several thicker flakes. Based on the comparison between experimental and theoretical SHG anisotropic patterns, the structural transition in the 5-layer SnSe is consistent with ferroelastic switching. Furthermore, we suggest the structural transition in thicker flakes results from partial ferroelastic switching, in which only a subset of layers undergoes a transition to a different ferroelastic state. Our observations should stimulate further experimental efforts toward the precise control of ferroelasticity in the MX family.

## 4 Methods

### 4.1 Sample Preparation

The SnSe single crystal was synthesized with the vertical Bridgman method. A 35 cm long quartz ampule with an outer diameter of 2 cm and an inner diameter of 1.8 cm was used to hold the tin and selenium elements, which had an equimolar ratio and a purity of 99.999%. The quartz ampule was first sealed at 10^–5^ Torr and then heated inside a furnace at a temperature of 600 °C for 50 h to homogenize the Sn and Se powders. Subsequently, the homogenized SnSe powder was smashed and loaded into the Bridgman furnace at 900 °C to allow the Sn and Se to completely melt, and the ampule was slowly drawn to a lower temperature region with a temperature gradient of about 0.1 mm/h. In the end, large SnSe single crystals were acquired after a period of 2 weeks, and the furnace was subsequently cooled down to room temperature, after which the obtained SnSe crystals were collected.

### 4.2 Raman Spectroscopy

The wavelength of the excitation laser is 532 nm. All measurements were conducted at room temperature. Raman spectra were acquired using a low excitation power of approximately 200–300 μW and short acquisition times (10–30 s) to minimize laser-induced heating and potential damage to the thin SnSe flakes. A 100× objective lens was used to focus the laser beam. Under these conditions, no visible degradation or spectral changes associated with laser heating were observed.

### 4.3 Polarization-Resolved SHG Measurements

The experimental setup for polarization-resolved SHG microscopy is outlined in Supplementary Figure S3. A Ti:sapphire oscillator operating at an 80 MHz repetition rate generated incident pulses centered at 800 nm. The laser power directed onto the samples was maintained at 150 μW for all measurements using a combination of a half-wave plate and a polarizing cube beamsplitter. This laser power was chosen based on preliminary tests to determine the maximum laser power that would avoid burning or damaging the flakes. A 50× objective lens with a numerical aperture (NA) of 0.5 was employed to focus the beam. The SHG light collected by the objective lens was then reflected by a dichroic mirror and detected by a photomultiplier tube (PMT). A bandpass filter was utilized to filter out the fundamental frequency. A raster scan of the incident beams with linear polarization was facilitated by galvo scanning mirrors, allowing for the generation of SHG intensity images. To selectively collect SHG photons with polarization either parallel or perpendicular to the incident polarization, a polarizer was positioned in front of the PMT. For angle-resolved SHG imaging, the sample was positioned on a rotational stage, enabling acquisition at intervals of 10°. The software ImageJ was utilized to analyze the mean SHG intensity from each SnS flake in the images.

### 4.4 Computational Methods

We conducted first-principles calculations using density functional theory implemented in the OpenMX code.^52^ The calculations employed the generalized gradient approximation (GGA),^53^ norm-conserving pseudopotentials,^54^ and optimized pseudoatomic basis functions.^55^ Specifically, we allocated three, two, and two optimized radial functions for the s, p, and d orbitals, respectively, for each Sn atom, with a cutoff radius of 9 Bohr (referred to as Sn9.0-s3p2d2). For Se atoms, we used S7.0-s3p2d2. In the case of the SnSe monolayer, a cutoff energy of 500 Ry was utilized for numerical integration. For numerical integrations and solving the Poisson equation in the self-consistent field (SCF) calculations, we employed a grid of dimensions 60 × 60 × 672 for all studies involving tensile and compressive strains in the SnSe monolayer, and 60 × 60 × 810 for the three-layer and five-layer SnSe. We used an 18 × 18 × 1 k-point mesh , and the effects of spin–orbit coupling are taken into account. The atomic forces were constrained to be smaller than 0.00005 Ha/Bohr for investigations into tensile strain in the SnSe monolayer, and 0.0001 Ha/Bohr for studies on compressive strain in the monolayer, as well as in the three-layer and five-layer SnSe. To calculate the SHG susceptibilities, we utilized momentum matrix elements based on the adopted pseudoatomic basis functions,^56–58^ and k-point samplings of 80 × 80 × 1 were employed for the monolayer, three layers, and five layers of SnSe, with a broadening parameter η = 0.1 eV. In the slab calculations, we selected a lattice constant of c = 50 Å for the monolayer and 60 Å for the three-layer and five-layer systems to mitigate interlayer interactions. The structures were fully relaxed initially without considering spin–orbit coupling. Subsequently, the atomic positions were relaxed again with spin–orbit coupling to simulate the monolayer’s structure, and the AB-stacked antiferroelectric (α-phase) structures of SnSe in three and five layers.

## Supplementary Material



## Notes

Additional powder XRD and pole figure XRD data; experimental setup and estimation of strain in AB-SnSe flakes; supplementary experimental results; electronic band structures of five-layer AB-SnSe before strain and under various tensile and compressive strains; SHG spectra of five-layer AB-SnSe before strain and under uniaxial and biaxial tensile strain; ferroelastic switching in monolayer SnSe and three-layer AB-SnSe under uniaxial tensile and compressive strain; and electronic band structures of monolayer SnSe and three-layer AB-SnSe under uniaxial tensile and compressive strain (PDF)
