# Supplementary material for: Structural Transition in Few-Layer Group-IV Monochalcogenides Induced by Mechanical Forces
Source: ACS Omega. 2026 May 19;11(21):31757–67. doi: 10.1021/acsomega.6c03304 (PMC13234651; doi:10.1021/acsomega.6c03304)
Supplement: Supplementary file 1 [file ao6c03304_si_001.pdf]

Supporting Information to

Structural transition in few-layer group-IV monochalcogenides induced by mechanical forces

Redhwan Moqbel[1], Krishna Ranganayakulu Vankayala [1], Rajesh Kumar Ulaganathan[2], Raman Sankar[1,4], Min-Nan Ou[1], Chi-Cheng Lee[3], Kung-Hsuan Lin[1,4,5]**

[1]Institute of Physics, Academia Sinica, Taipei 115201, Taiwan

[2]Centre for Nanotechnology, Indian Institute of Technology Roorkee-247667, India.

[3]Department of Physics, Tamkang University, Tamsui Dist., New Taipei 251301, Taiwan

[4]Taiwan Consortium of Emergent Crystalline Materials, Ministry of Science and Technology, Taipei 106214, Taiwan

[5]Graduate Institute of Applied Physics, National Chengchi University, Taipei 116026, Taiwan.

*Email of Corresponding authors: linkh@sinica.edu.tw, and cclee.physics@gmail.com.

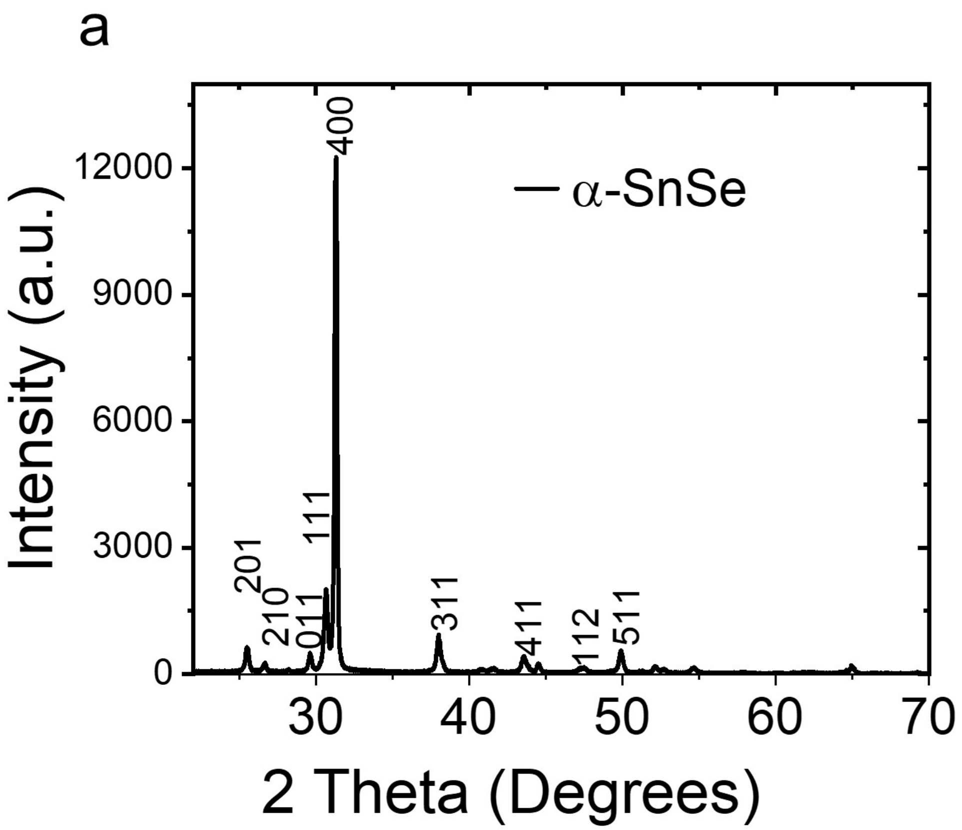

Figure S1: (a) Powder X-ray diffraction XRD Patterns of α-SnSe prepared by CVT.

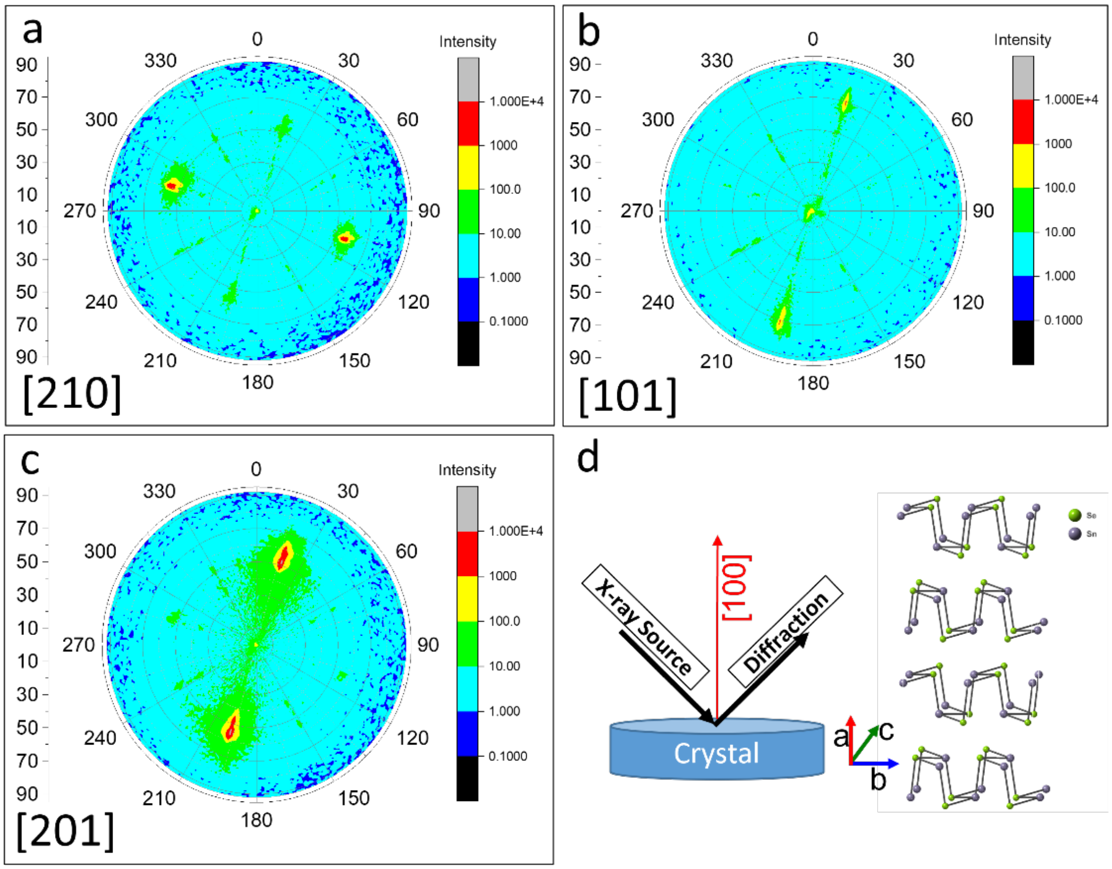

Figure S2: The full-scanning of (a) [210], (b) [101], and (c) [201] with pole (100). (d) Schematics of the lattice structure and space-relations of lattice axes to the XRD scanning plane.

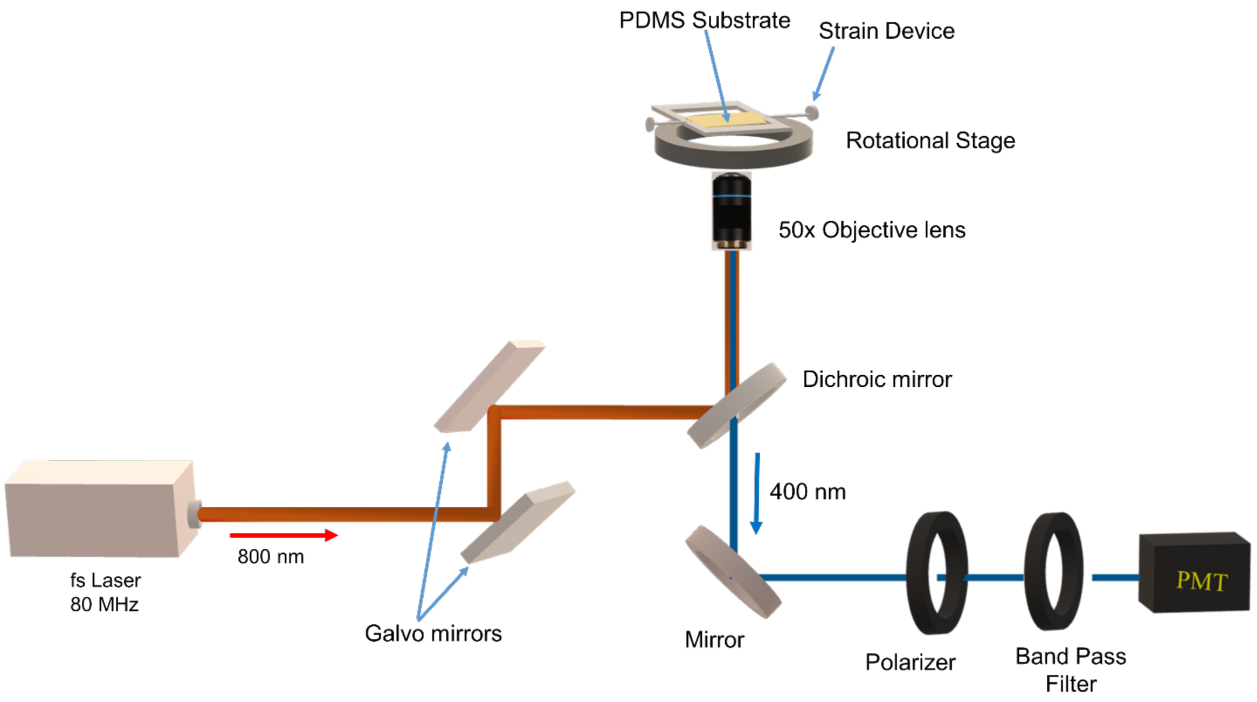

Figure S3: Experimental setup of SHG microscopy.

Note 1 Evaluation of strain exerted on the SnSe flakes.

The strain ε is calculated as follows: $\varepsilon = [(L' - L)/L] \times 100\%$ where L' is the length after strain, and L is the length before strain. While applying strain on the PDMS substrate, the actual strain transferred to the samples on the PDMS substrate can be evaluated by measuring the deformation of the samples. However, the SnSe flakes on the PDMS substrate typically measure around 300 nm, which is too small to analyze the deformation length. To tackle this challenge, we utilized larger exfoliated layers of InSe on a PDMS substrate and measured the deformation of InSe. By this method, we estimated the strain transfer rate from the PDMS to the samples on top and estimated the strain of SnSe flakes. While the estimated strain might be also slightly overestimated by this method, the values are much closer to the actual strain on SnSe compared with the strain on the PDMS substrate. **Figure S4** shows the optical microscope images of InSe flakes when the strain applied on the PDMS substrate from 0% to 30.63%. The red lines in each image measure the lengths of a flake along the strain direction. **Figure S4f** illustrates the relationship between the strain on the PDMS substrate and the strain on the InSe flake. By using these figures, we estimated the actual strain on the SnSe flakes according to the strain of the PDMS substrate.

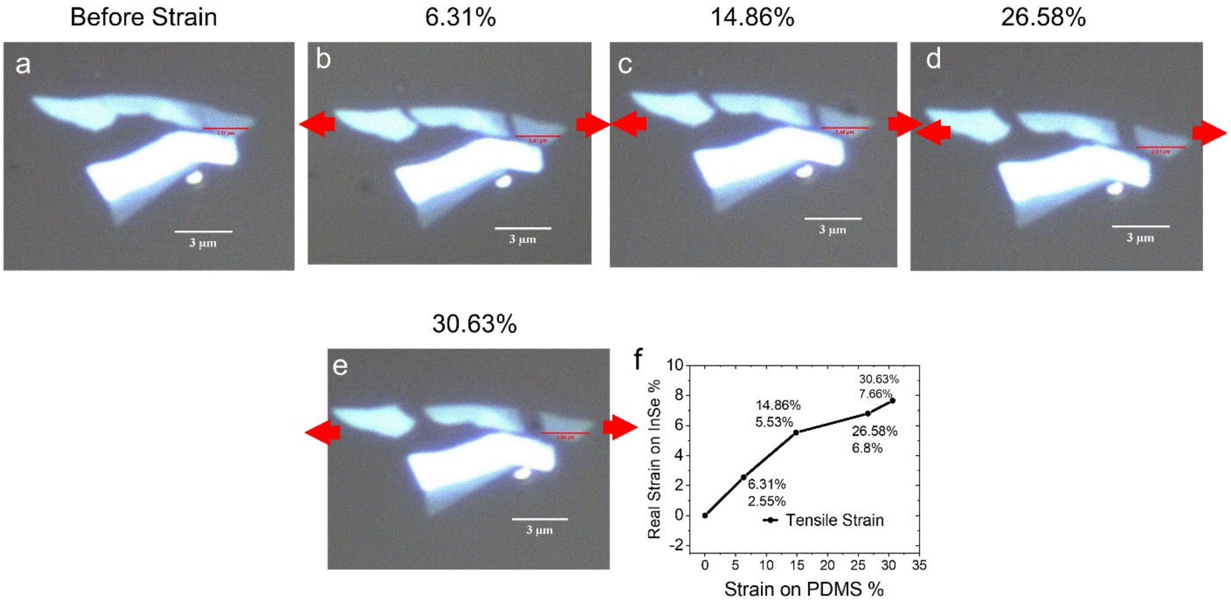

Figure S4: Optical microscope images of InSe flakes when the PDMS substrate is applied with strain of (a) 0%, (b) 6.31%, (c)14.86%, (d)26.58%, and (e)30.63%, respectively along the direction indicated by the red arrows. (f) Relationship between the strain applied on the PDMS and the actual strain experienced by the InSe flake

Note 2 Partial ferroelastic switching in the SnSe flakes

Additional investigations on two other SnSe flakes yielded similar results (**Figures S5 and S6**). In **Figure S5e**, mechanical strain induces a four-lobed SHG polar pattern, consistent with the simulated partial switching shown in **Figure 7h**. A distinct behavior was observed in the SnSe sample shown in **Figure S6**. The initial SHG polar response displayed the same typical two-lobed structure. However, after multiple strain–release cycles, the SHG polar pattern evolved into a six-lobed configuration (**Figure S6e**). The six-lobes may be the superposition of the four-lobed pattern and the initial two-lobed structure, resulting from another complicated layered structures with partial ferroelastic switching.

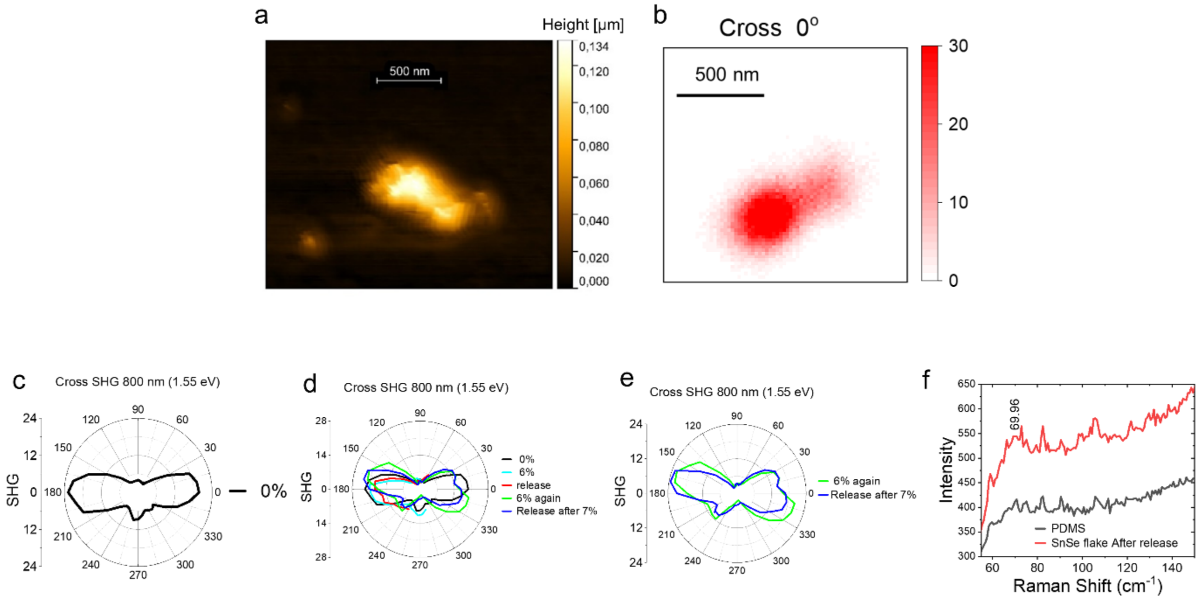

Figure S5: (a) AFM image of the SnSe flake on PDMS substrate. (b) SHG images taken at an angle of 0°. Cross SHG polar patterns are shown (c) before strain, and (d) after applying varying levels of tensile strain along the x-axis (zigzag), and release. (e) Cross SHG polar patterns are

shown after 6% tensile strain in the x-axis and after release from 7% strain. (f) Raman spectrum of the SnSe flake in AFM and SHG images.

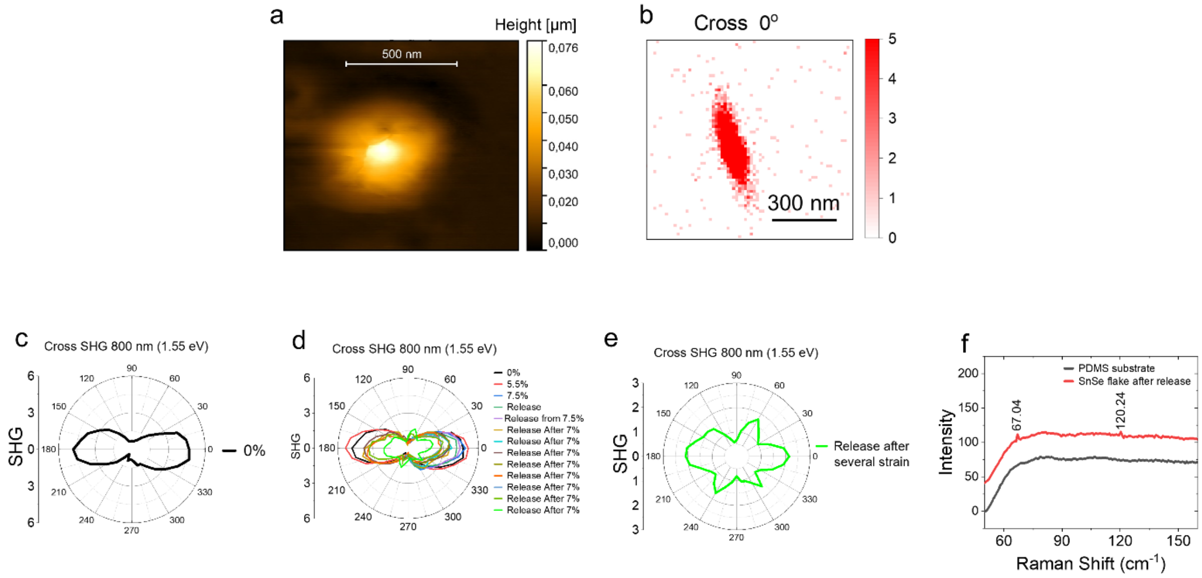

Figure S6: (a) AFM image of the SnSe flake on PDMS substrate. (b) SHG images taken at an angle of 0°. Cross SHG polar patterns are shown (c) before strain, and (d) after applying varying levels of tensile strain along the x-axis (zigzag), and release. (e) Cross SHG polar patterns are shown after release from several strains in the x-axis. (f) Raman spectrum of the SnSe flake in AFM and SHG images.

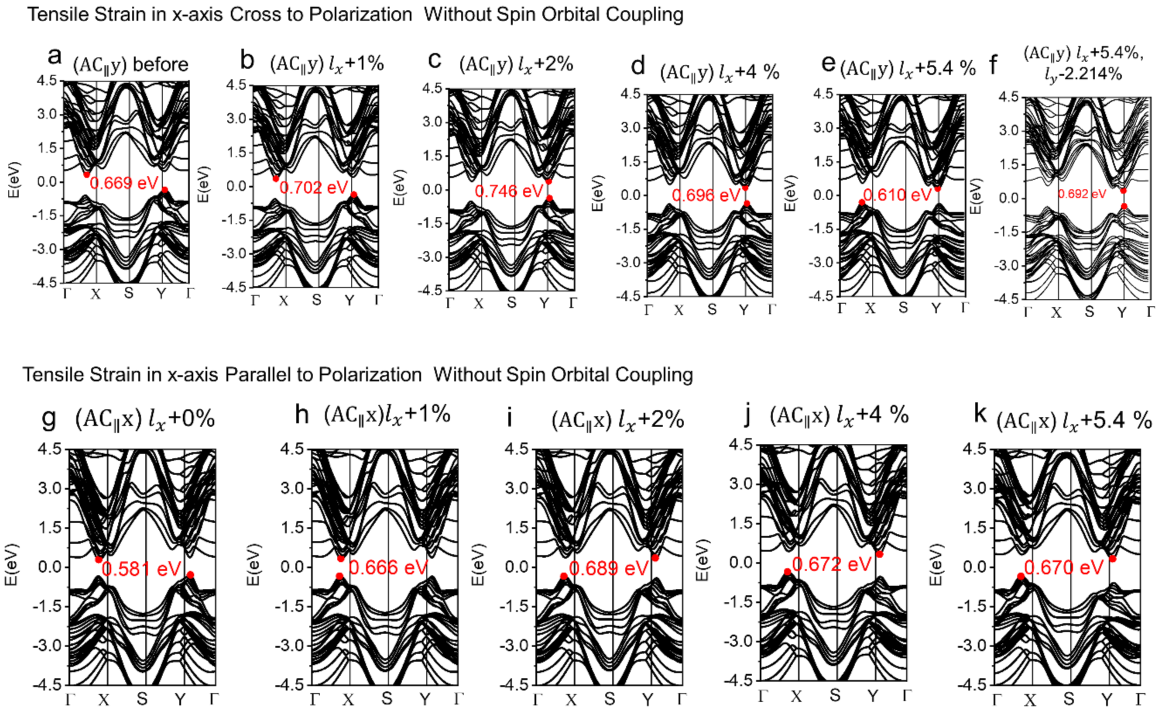

Figure S7: Energy band structure of AB-SnSe five layers under different strain conditions.

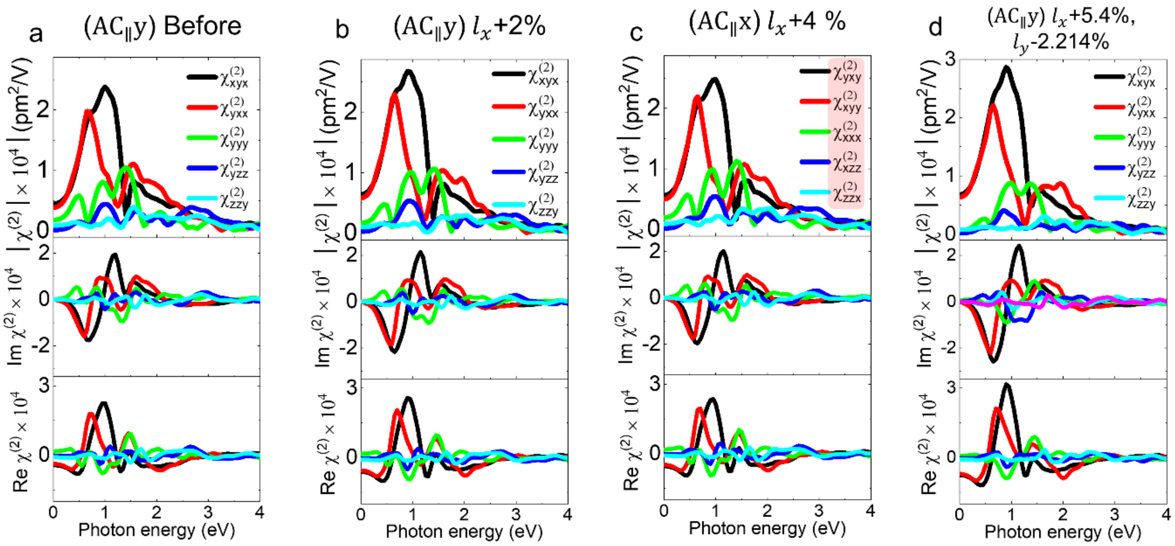

Figure S8: SHG spectra of AB-SnSe five layers for crystal orientations (a) ($AC_{\parallel}$y) without strain, (b) ($AC_{\parallel}$y) with 2% uniaxial tensile strain along x, (c) ($AC_{\parallel}$x) with 4% uniaxial tensile strain along x, and (d) ($AC_{\parallel}$y) with biaxial strain (5.4%, -2.214%) along (x, y). The red shadow in c highlight the different nonzero susceptibilities after ferroelastic switching from ($AC_{\parallel}$y) to ($AC_{\parallel}$x).

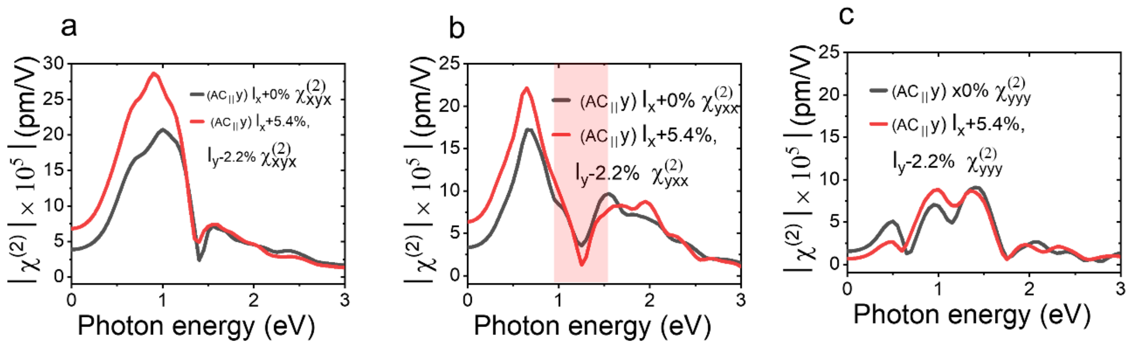

Figure S9: Absolute values of the primary three SHG susceptibility spectra unstrained and strained AB-SnSe five layers for comparison. At 1.55 eV, the difference in polar plots of Fig. 4(e) in the main text primarily result from the changes of $\chi_{yxx}^{(2)}$ as shown in (b).

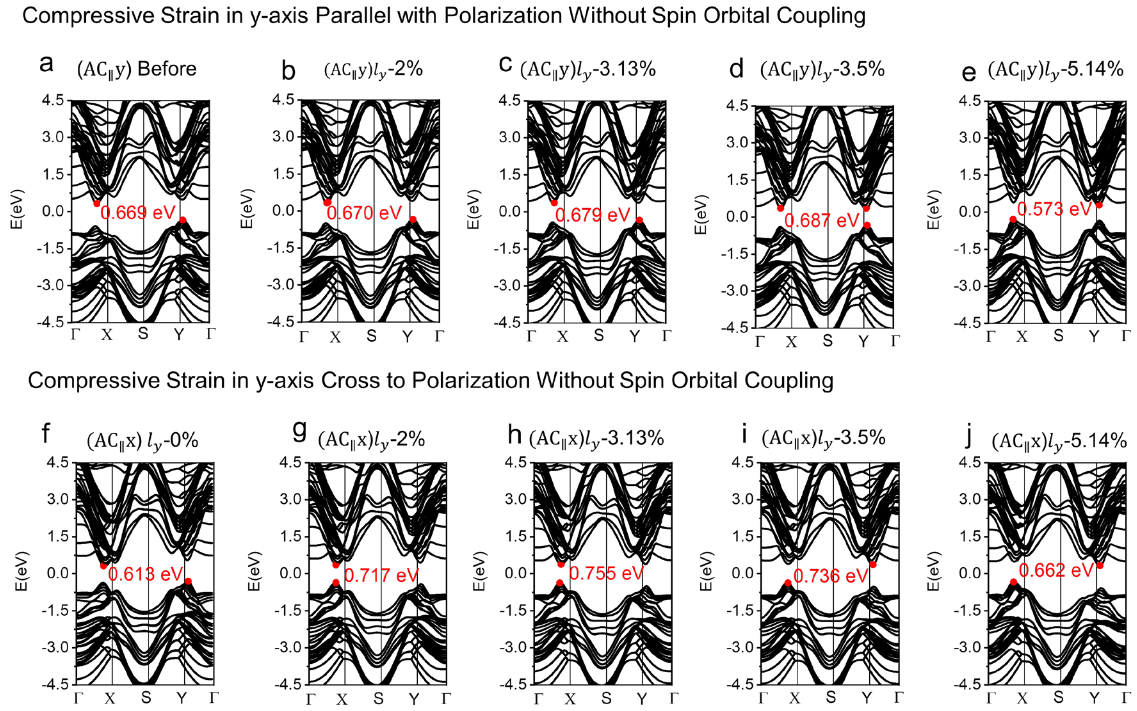

Figure S10: Energy band structure of AB-SnSe five layers under compressive uniaxial strain along y.

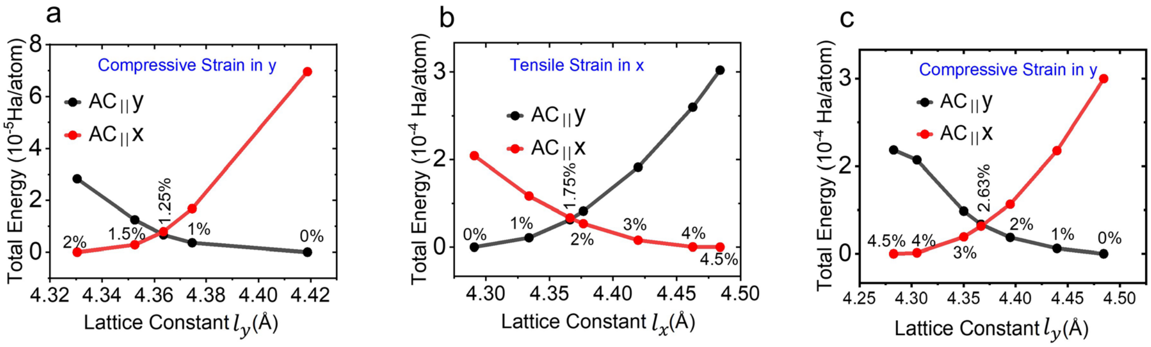

Figure S11: Calculated total energy of (a) SnSe monolayer versus compressive uniaxial strain along y, (b) three-layer AB-SnSe versus tensile uniaxial strain along x, and (c) three-layer AB-SnSe versus compressive uniaxial strain along y.

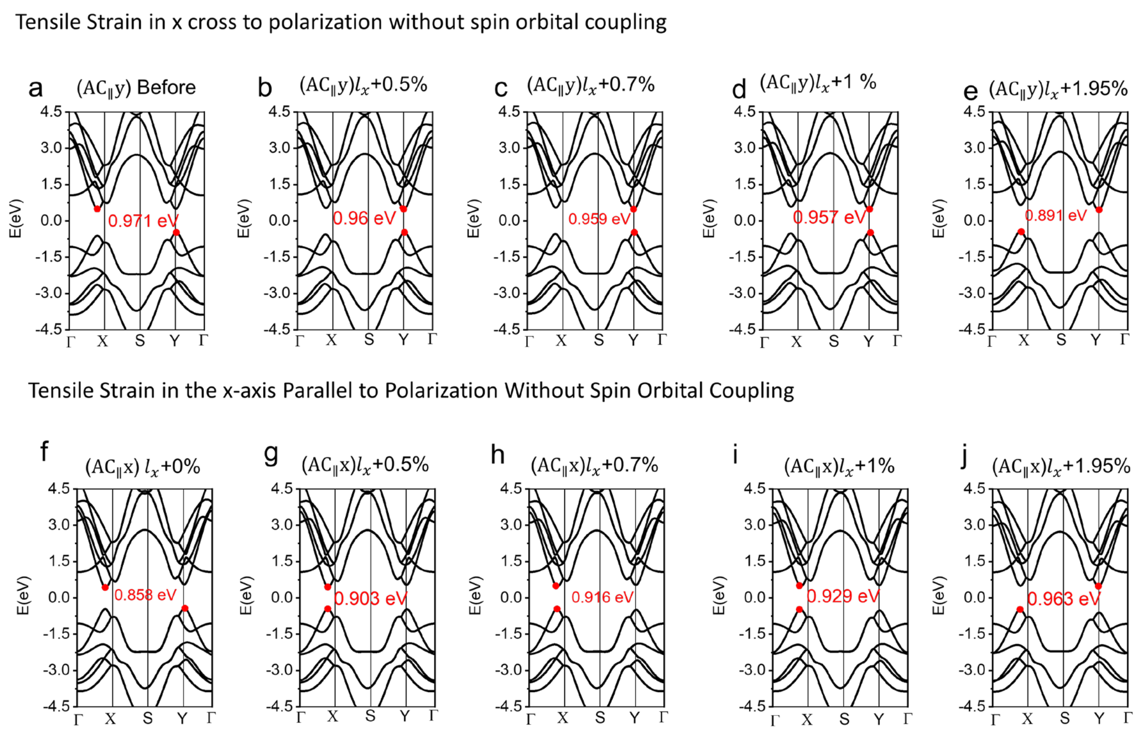

Figure S12: Energy band structure of SnSe monolayer under tensile uniaxial strain along x.

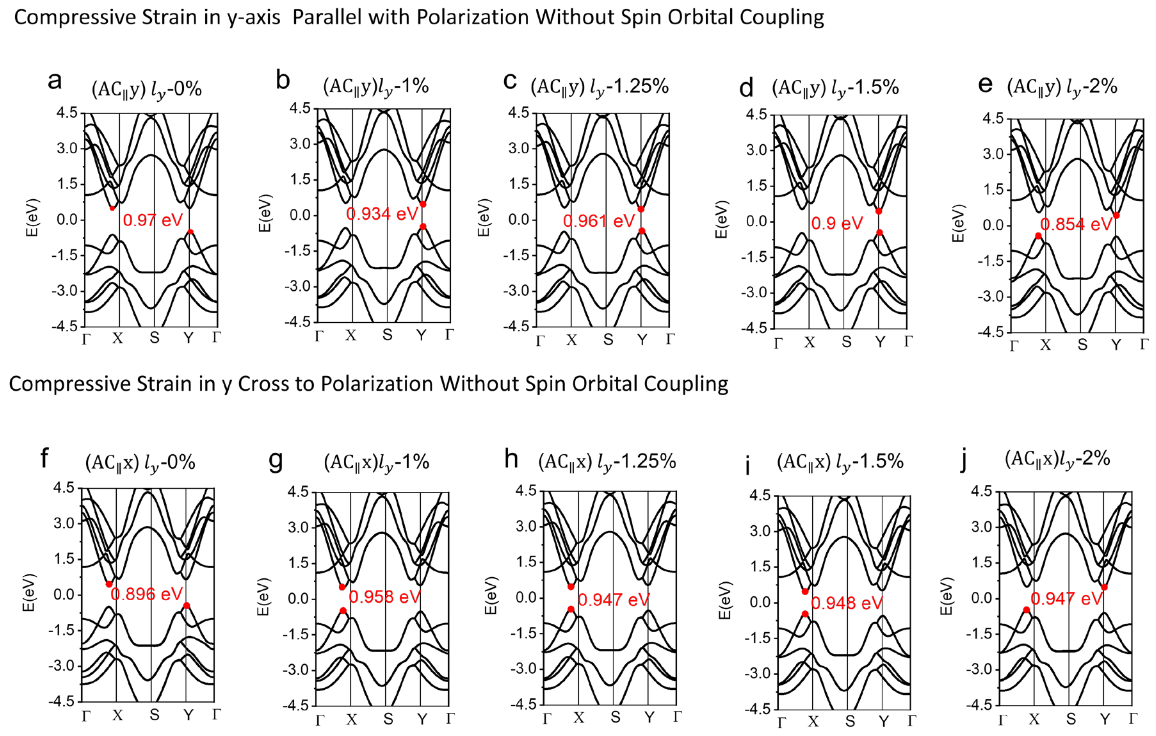

Figure S13: Energy band structure of SnSe monolayer under compressive uniaxial strain along y.

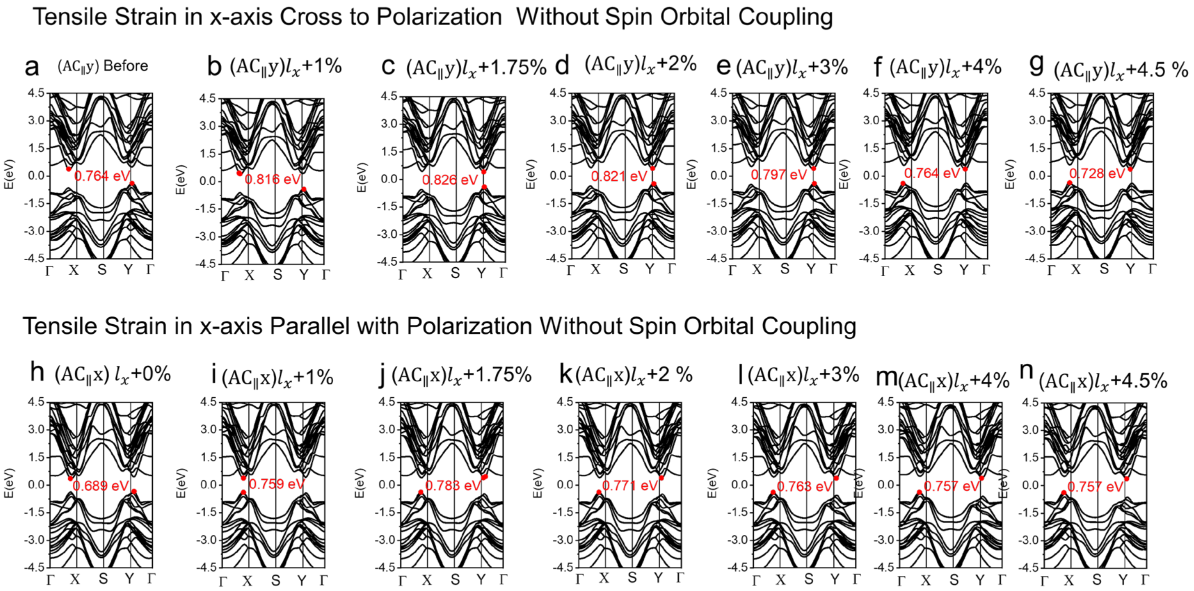

Figure S14: Energy band structure of three-layer AB-SnSe under tensile uniaxial strain along x.

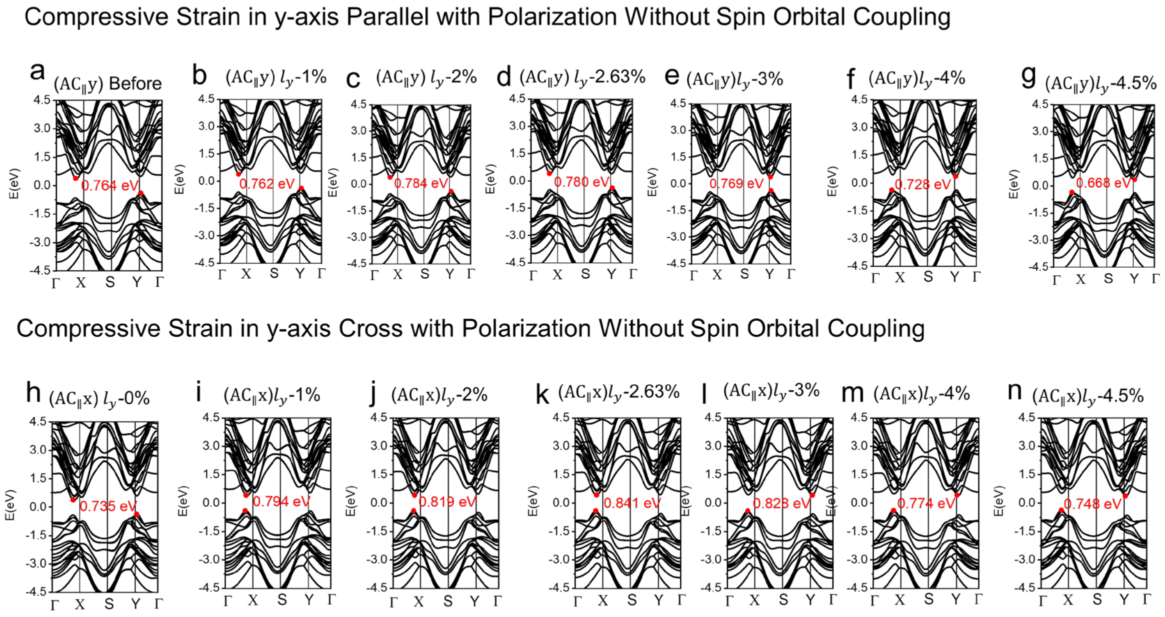

Figure S15: Energy band structure of three-layer AB-SnSe under compressive uniaxial strain along y.
